# Cyclic changes in cortisol across the estrous cycle in parous and nulliparous Asian elephants

**DOI:** 10.1530/EC-14-0025

**Published:** 2014-04-15

**Authors:** Kerry V Fanson, Tamara Keeley, Benjamin G Fanson

**Affiliations:** 1 Wildlife Reproductive Centre, Taronga Conservation Society Australia Dubbo, New South Wales Australia; 2 School of Life and Environmental Sciences, Deakin University 75 Pigdons Road, Waurn Ponds, Victoria, 3217 Australia; 3 School of Agriculture and Food Sciences, University of Queensland Gatton, Queensland Australia

**Keywords:** adrenal, glucocorticoids, ovarian, Proboscidea, reproduction

## Abstract

In the context of reproduction, glucocorticoids (GCs) are generally considered to have negative effects. However, in well-studied model species, GCs fluctuate predictability across the estrous cycles, and short-term increases promote healthy ovarian function. Reproductive challenges have plagued captive elephant populations, which are not currently self-sustaining. Efforts to understand reproductive dysfunction in elephants have focused on the suppressive effects of cortisol, but the potential permissive or stimulatory effects of cortisol are unknown. In this study, we provide a detailed examination of cortisol patterns across the estrous cycle in Asian elephants (*Elephas maximus*). Time series analysis was used to analyze cortisol and progesterone data for a total of 73 cycles from eight females. We also compared cortisol profiles between females that successfully conceived and females that failed to conceive despite repeated mating attempts. Our results revealed that cortisol fluctuates predictably across the estrous cycle, with a peak during the second half of the follicular phase followed by low levels throughout the luteal phase. Furthermore, this pattern was significantly altered in nulliparous females; cortisol concentrations did not decline during the luteal phase to the same extent as in parous females. This study highlights the complexity of cortisol signaling and suggests future directions for understanding the role of cortisol in reproductive dysfunction.

## Introduction

Elevated glucocorticoid (GC) titers associated with chronic stress can lead to reproductive failure (1). Consequently, studies of reproductive dysfunction often focus on the suppressive effects of GCs. However, GCs also play a critical role in the promotion of healthy reproductive function (reviewed in (2, 3, 4, 5)). Elevations in GCs during proestrus have been shown to promote receptive behavior, stimulate gonadotropins, facilitate ovulation, and reduce damage caused by inflammation (4, 6, 7, 8, 9, 10). In contrast, disruption of these GC patterns can lead to impaired follicle development and irregular estrous cycles (4, 11, 12). Therefore, under normal physiological levels, short-term increases in GCs can have a positive effect on reproductive function.

Captive elephants have notoriously poor reproductive function, and the North American population is not self-sustaining (13, 14, 15, 16). With the re-accelerated loss of elephants in the wild, elephant conservation has been identified as one of the top ten global conservation issues (17). Captive breeding programs play a critical role in achieving this goal, but fertility rates must improve. Causes of ovarian dysfunction and reproductive failure in elephants are poorly understood, despite the numerous studies on elephants with both normal and abnormal estrous cycles (18, 19, 20, 21, 22, 23, 24, 25, 26, 27, 28, 29, 30, 31). Longitudinal profiles across the estrous cycle have been published for several hormones, including estradiol (E_2_), progestagens, luteinizing hormone (LH), follicle-stimulating hormone, prolactin, and inhibin (19, 20, 21, 28, 29).

Chronic stress was one of the first theories proposed for ovarian dysfunction in elephants, but previous studies have not found the evidence that mean cortisol levels differ between cyclic and acyclic females (21, 24). However, this does not rule out the possibility that more fine-scale patterns of cortisol expression are disrupted in non-cycling elephants. Mean hormone values provide limited information, and it is often the pattern of hormone secretion that is more biologically relevant (32). There is some evidence that cortisol titers increase during the follicular phase in cycling African elephants (18). However, the potential role of cortisol in promoting healthy ovarian function in elephants has not garnered much attention.

The goal of this study was to establish a foundation for understanding the role of cortisol in ovarian function in Asian elephants (*Elephas maximus*). In concert with progesterone data, we present an extensive examination of cortisol patterns across the estrous cycle in both parous and nulliparous females (Table 1). First, we characterized the broad patterns of cortisol expression relative to progesterone in eight females that exhibited regular estrous cycles (based on progesterone data). We analyzed a total of 73 cycles using time series analysis, which provides a powerful tool for detecting cyclic patterns in longitudinal datasets. Second, we established a more detailed ‘normative’ cortisol profile using data from females that subsequently became pregnant (parous females; *n*=6). Third, we compared this normative cortisol profile with i) profiles from nulliparous females (*n*=2) and ii) data collected after a long-distance transfer (*n*=6). Finally, we propose a novel hypothesis for understanding the role of cortisol in elephant reproduction.

## Materials and methods

### Study animals and sample collection

This study included eight adult female Asian elephants housed at Taronga Zoo (Sydney, NSW, Australia) and Melbourne Zoo (Melbourne, VIC, Australia; Table 1). Females ranged in age from 5 to 33 years old at the start of the study. All females exhibited regular ovarian cycles based on progesterone profiles. Females had limited access to a mature bull elephant, so mating attempts were either via monitored introductions or artificial insemination (AI). Six females successfully conceived on the first attempt, further demonstrating that these females exhibited healthy reproductive function. Two females failed to conceive, despite repeated mating opportunities. One of the females (F7) had scheduled introductions to a mature male on the days surrounding ovulation (as determined from hormone monitoring). These introductions occurred every cycle for the last 3 years of the study (ten cycles), with the exception of one cycle when AI was attempted. Since the study concluded, she has continued to have regular mating introductions and a second AI attempt, but she has still not conceived. The oldest female (F3) was housed with a male at least 50% of the time for 28 years (until the arrival of the other females from Thailand). She was diagnosed with uterine tumors shortly before the initiation of the study. A gonadotropin-releasing hormone (GnRH) antagonist was used to suppress ovarian activity in this female 1.5 years into the study. Only samples collected before administration of the GnRH antagonist were included in the data analysis.

**Figure 1:**
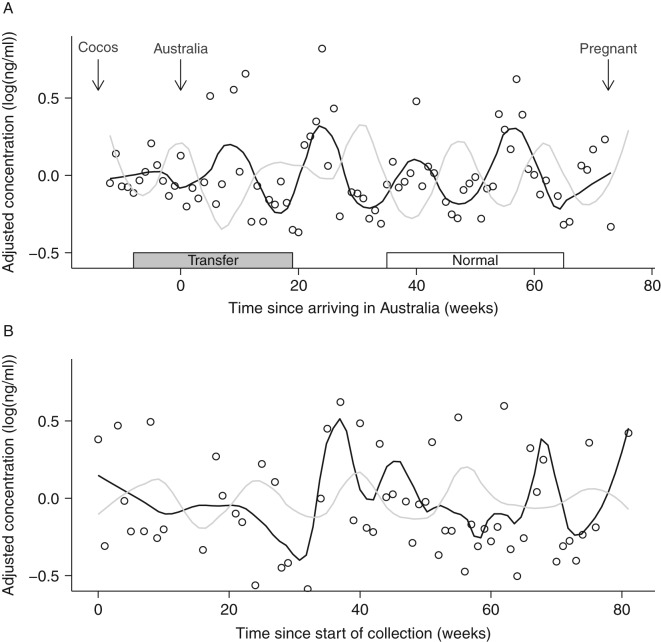
Representative longitudinal hormone plots for (A) a parous female Asian elephant (F1) and (B) a nulliparous female (F3; not transferred from Thailand). Lines represent loess smoothing curves for cortisol (black) and progesterone (gray). Circles represent individual cortisol data points. Hormone concentrations have been detrended (see text), and thus data are expressed as residual values. Arrows represent the timing of transfer events and pregnancy. Boxes at the bottom indicate windows of time used to establish normative hormone patterns (‘normal’) and test for an effect of transfer (‘transfer’).

The youngest seven elephants were transferred from Thailand to Australia via Cocos Keeling Island at the beginning of the study (Fig. 1; also see (33) regarding adrenal response to transfer). Sample collection was initiated on Cocos Keeling Island 3 months before the elephants were moved to mainland Australia. Following the transfer, there was a gap in sample collection (∼1 month), so this dataset does not reflect acute stress associated with transfer. Based on progesterone data, all females continued to cycle at regular intervals throughout the transfer.

Serum samples were collected approximately once per week over a period of 7 years (2006–2012). Only data before an elephant's first pregnancy (or GnRH antagonist for F3) were included. Therefore, the number of complete cycles monitored for each elephant ranged from 4 to 16 (Table 1). Elephants were conditioned for routine blood collection from ear veins. Blood samples were collected in late morning and centrifuged to separate serum. All samples were stored at −20 °C and shipped to the Wildlife Reproductive Centre at Taronga Western Plains Zoo for analysis.

### Hormone analysis

Antibodies and the corresponding HRP conjugate were obtained from C. Munro (University of California, Davis, CA, USA). Enzyme-immunoassay protocols are similar to those previously described (33, 34). Briefly, 96-well microtiter plates (Nunc Maxisorp, In Vitro Technologies, Melbourne, Australia) were coated with 50 μl antibody solution and incubated overnight at 4 °C. Plates were washed to remove unbound antibody, and 50 μl of standard, control, or sample plus 50 μl of HRP-conjugate were immediately added to each well. After incubating for 2 h at room temperature, plates were washed and 100 μl of substrate solution was added to each well. Absorbance was read at 405 nm using an optical density plate reader (Dynex MRX Revelation, Dynex Technologies, Chantilly, VA, USA). To monitor precision and reproducibility, low (70% binding) and high (30% binding) control samples were run on each plate.

Progesterone was quantified using the monoclonal progesterone CL425 antibody previously validated for elephants (35, 36). The antibody had the following cross-reactivities: 100% progesterone, 55% 5α-pregnen-3,20-dione and <0.1% pregnanediol, androstenedione, and corticosterone. Assay sensitivity was 0.05 ng/well. Intra-assay coefficients of variation (CVs) were 11.01 and 3.44% (*n*=13) for low and high controls respectively. The inter-assay CVs were <15%.

Cortisol was quantified using the cortisol R4866 antibody previously validated for elephant serum (33). The antibody had the following cross-reactivities: cortisol 100%, prednisolone 9.9%, prednisone 6.3%, cortisone 5%, and <1% with corticosterone, desoxycorticosterone, 21-desoxycortisone, testosterone, androstenedione, androsterone, and 11-desoxycortisol. Assay sensitivity was 0.08 ng/ml. Intra-assay CVs were 6.3 and 3.4% (*n*=17) for low and high controls, respectively, and inter-assay CVs were <15%.

### Statistical analysis

Statistical analyses were conducted using SAS v9.2 (Cary, NC, USA). For all analyses, hormone concentrations were log-transformed, averaged by week and then detrended for each elephant and hormone type. Detrending is a standard component of time series analysis, and serves to remove long-term trends in the data that may exaggerate correlations between datasets. For example, one elephant exhibited a steady increase in both progesterone and cortisol concentrations over time. This would produce a positive correlation between hormones, regardless of patterns within estrous cycles. Detrending was performed by fitting a cubic linear model to the hormonal concentrations in order to remove trends over time. Graphical analysis of the residuals indicated satisfactory removal of non-cyclic trends of the data. Residuals were then used for all subsequent analyses.

#### Characterization of cortisol cycles

Time series analysis was used to test for cycles in the cortisol data. This analysis included all cycles for all eight females (total=73 cycles). Time series analysis becomes more robust with increasing length of the dataset; therefore we did not exclude cycles surrounding the transfer. All females continued to exhibit regular estrous cycles (based on progesterone data) throughout the transfer. Acute responses to the transfer (1 month post-transfer) were not included.

**Figure 2:**
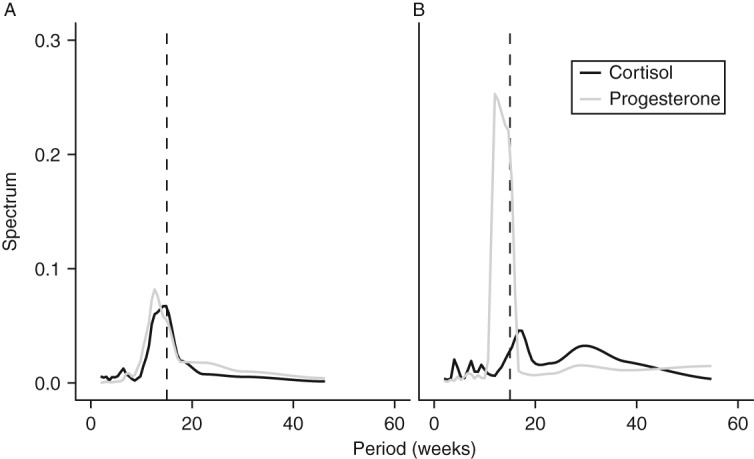
Spectral analysis plot for identifying cyclic patterns in two representative elephants. (A) F1 shows distinct peak at frequencies near 15 weeks (indicated by dashed line) for both cortisol and progesterone. (B) F2 has a distinct progesterone peak near 15 weeks and two modest peaks for cortisol, one around 15 weeks and another near 30 weeks.

We used spectral analysis (37) to identify cycles and determine cycle frequency (duration) for both progesterone and cortisol. Briefly, spectral analysis provides a tool for identifying dominant cycle frequencies within a dataset, with higher spectrum values indicating the presence of a cycle. As expected, all elephants had a distinct spectral peak for progesterone data around 15 weeks (Fig. 2).

Cross-spectrum spectral analysis expands upon this analysis by allowing the comparison of two sets of time series data (in this case, the two different hormones). Squared-coherency scores were used to assess whether the cycle periodicity of cortisol and progesterone was significantly aligned within each individual (i.e. how similar are cycle lengths for these two hormones). Similar to a correlation, squared-coherency scores range from 0 (no association) to 1 (perfect association). Significance was determined by constructing a 95% CI around the peak coherency value for each elephant and assessing whether this CI included 0.

Finally, to visualize the alignment of progesterone and cortisol cycles, we conducted a regression analysis for time series data, in which we fitted a sinusoidal function to each hormone for each individual (37). For the time series regression, the model included sinusoidal components of the cycle (sine and cosine) which estimate the amplitude and phase angle of the wave (i.e. how waves are aligned). Average estrous cycle duration (15 weeks) was defined as the period length/cycle frequency. The error term was modeled using a second-order autoregressive structure to account for the fact that data that are closer together in time are more correlated. A Barlett's Kolmogorov–Sirnov test of the residuals indicated that the correlation structure of the model was sufficient.

#### Normative changes in cortisol across the estrous cycle

There were six females in the study which successfully conceived, indicating that they had healthy patterns of ovarian function. To characterize the ‘normative’ patterns of hormone expression, we selected the two cycles before the cycle in which the female conceived (see Fig. 1). To avoid the confounding effects of implantation and pregnancy, we did not include the cycle in which the female became pregnant. Cycles were aligned by calculating the time from ovulation (week 0). Ovulation was estimated using known LH peaks and post-ovulatory increases in progesterone (concentrations doubled between subsequent samples and continued to increase). The follicular phase preceding and the luteal phase following ovulation was considered one full cycle. Data were averaged within and across females.

#### Cortisol profiles for nulliparous and transferred females

For the two nulliparous females, we selected cycles surrounding times when mating was attempted but the female failed to become pregnant (F3=five cycles and F7=11 cycles). Mating attempts included continuous housing with a mature male (F3) and scheduled introductions to a mature male or AI (F7). We compared progesterone and cortisol profiles between ‘nulliparous’ and ‘normative’ cycles (described above) using separate linear mixed models for each hormone. Models tested for the effect of time (week of estrous cycle) and status (nulliparous or parous) on mean hormone levels. Models also included an interaction term (week×status) to examine whether patterns of hormone expression varied between parous and nulliparous females. Since week is a repeated factor, we included female ID as a random factor in the model. We performed contrast statements to specifically test for differences between parous and nulliparous females during i) the second follicular wave when cortisol is elevated (weeks −3 to 0, between the anovulatory and ovulatory LH peaks) and ii) the luteal phase when cortisol is typically low (weeks 2–5).

To assess whether the transfer impacted broad (i.e. not acute) patterns of hormone expression, we compared ‘transfer’ and ‘normative’ profiles of the six parous females. For transfer data, we selected the first two complete cycles for which we had data (see Fig. 1). The first cycle generally straddled the move to Australia, and the second cycle occurred in Australia. The cycles used for ‘normative’ profiles are as described earlier (two cycles before conception). Linear mixed models tested for an effect of a week of estrous and cycle type (transfer or normal) and their interaction on mean hormone levels. Female ID was included as a random effect. Similar to the nulliparous analysis, contrast statements were performed to test for differences during the second follicular wave and luteal phase.

## Results

### Characterization of cortisol cycles

For all eight females, graphical analysis revealed cyclic patterns in cortisol (e.g. Fig. 1). Profiles for five females (F1, F4, F5, F6, and F8) were similar to the profile shown in Fig. 1a, with a peak in cortisol occurring between progesterone peaks. Females F2 and F7 had some cycles that were similar to Fig. 1a, and some cycles where the cortisol peak was either missing or shifted. Female F3, who had uterine tumors, had the least apparent cycles in cortisol.

**Figure 3:**
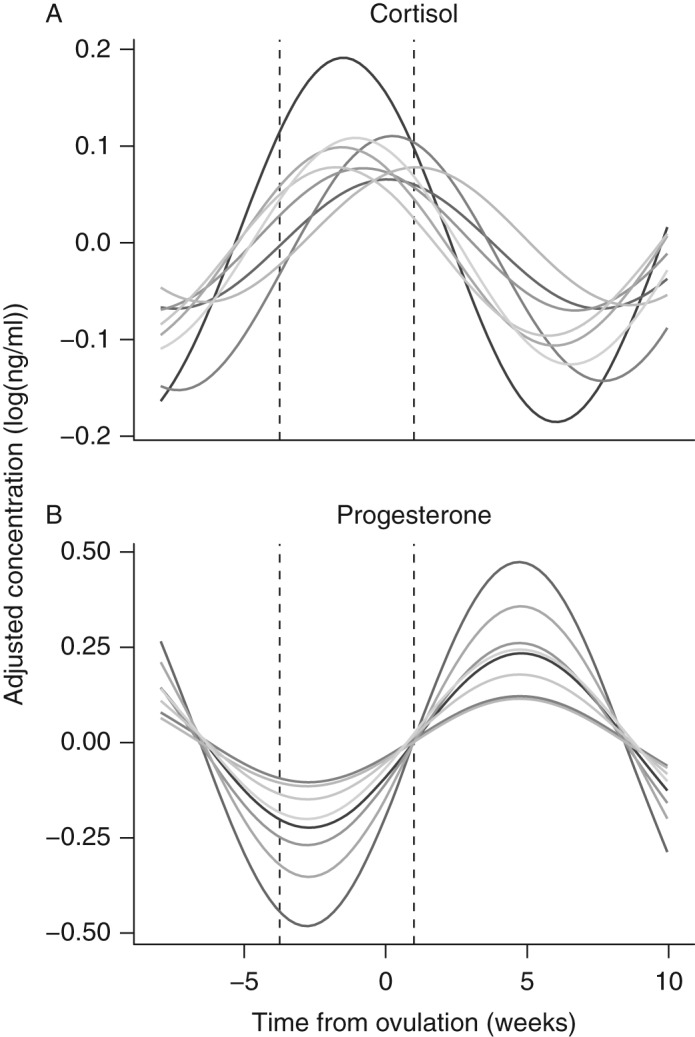
Predicted cycles for (A) cortisol and (B) progesterone obtained from time series regression analysis. Lines represent individual Asian elephants. Despite variation in cortisol data among the elephants, cortisol cycles consistently peak between the trough and inflection point of the progesterone cycles (vertical dotted lines).

Spectral analysis indicated that for seven females (except F3), the dominant frequency (duration) of these cortisol cycles was 15 weeks (Fig. 2). This corresponds with the dominant frequency for progesterone cycles, which was also 15 weeks. Cross-spectrum spectral analysis confirmed that cycle periodicity for progesterone and cortisol was significantly correlated for six females (Table 2, ‘coherency’). This shows that progesterone and cortisol cycled with similar frequency within most females. Cortisol cycles were shifted ∼130° in front of progesterone cycles, indicating that cortisol peaked during the second half of the follicular phase (Fig. 3).

### Normative changes in cortisol across the estrous cycle

**Figure 4:**
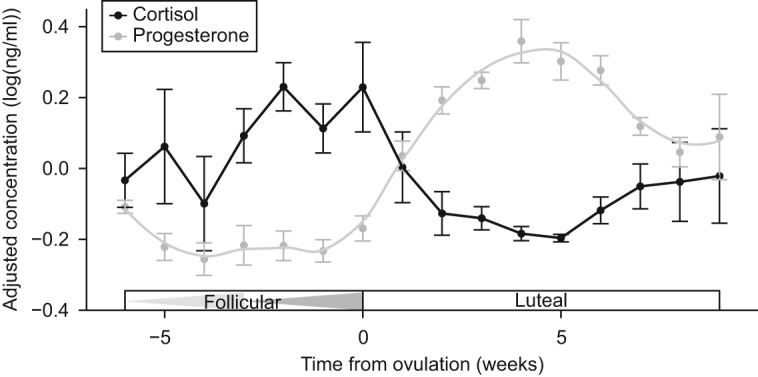
Normative cortisol and progesterone patterns for parous Asian elephants across the estrous cycle. Data represent mean±1 s.e.m. for two cycles preceding conception (*n*=6 females). Triangles in the 'Follicular' box indicate the first and second follicular waves. Data represent mean±1 s.e.m., and are expressed as detrended residuals (see text).

To take a closer look at changes in cortisol across the estrous cycle, normative cortisol patterns were established using data from six parous females (Fig. 4). Analyses included data from the two full cycles preceding a female's first conceptive cycle (Fig. 1). Concentrations increased during the first half of the follicular phase and remained elevated during the second half of the follicular phase. There was another peak at ovulation, after which concentrations fell and remained low for most of the luteal phase.

### Cortisol profiles for nulliparous and transferred females

**Figure 5:**
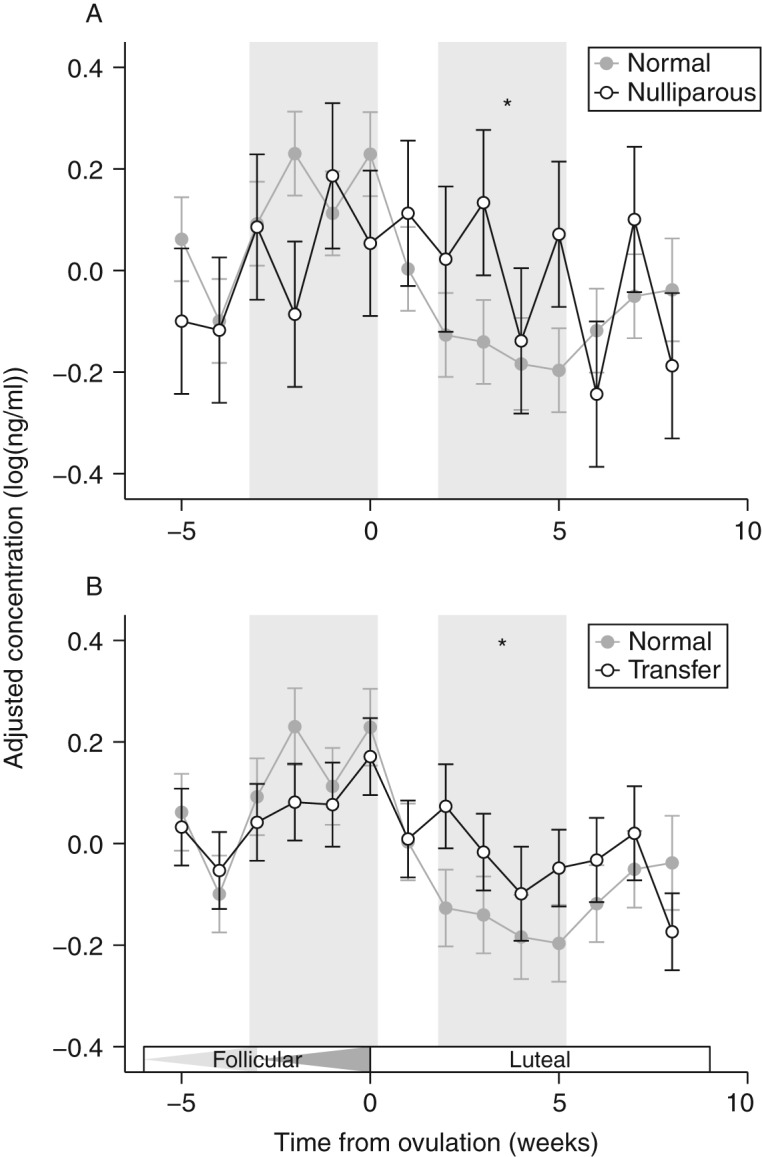
Cortisol patterns for (A) nulliparous females and (B) females undergoing long-distance transfer. In both graphs, ‘normal’ cortisol patterns (from Fig. 4) are shown in gray for comparison. Data represent mean±1 s.e.m., and are expressed as detrended residuals (see text). Comparisons between groups were made at two time points (indicated by gray bars): the second follicular wave (first bar) and the luteal phase (second bar). Triangles in the 'Follicular' box indicate the first and second follicular waves. Asterisks indicate significant differences between the groups (*P*<0.05).

Two females in the study failed to conceive despite repeated mating efforts (either natural or assisted). Although both nulliparous females had regular progesterone cycles, their infertility suggests that other aspects of reproductive function may have been compromised. During the follicular phase, cortisol titers for nulliparous females did not differ significantly from parous females (Fig. 5A; *t*_75_=−1.28, *P*=0.20). However, cortisol titers for nulliparous females did not decrease after ovulation and were significantly higher than parous females during the luteal phase (Fig. 5A; *t*_75_=2.22, *P*=0.03). Progesterone patterns were not significantly different between parous and nulliparous females (*F*_13,75_=0.86, *P*=0.60).

Seven females were transferred from Thailand to Australia at the beginning of the study (33). For the six parous females, we conducted a within-female comparison (‘transfer’ vs ‘normative’; see Fig. 1 for sampling intervals) to examine whether long-distance transfer affected broad patterns of cortisol expression (acute effects of transfer were not included). Similar to the results for nulliparous females, pre-ovulatory (follicular) cortisol levels were not significantly different (Fig. 5B; *t*_108_=1.24, *P*=0.22). However, during the luteal phase, ‘transfer’ cortisol titers did not decrease to the same extent as normative profiles (Fig. 5B; *t*_108_=2.24, *P*=0.03). Progesterone profiles during the transfer did not differ from normative cycles (*F*_13,102_=0.61, *P*=0.84).

## Discussion

### Normative cortisol patterns

We show that circulating cortisol concentrations fluctuate in a distinct cyclic manner across the estrous cycle in Asian elephants. The cortisol cycle is shifted ∼130° in front of the progesterone cycle, such that the crest occurs during the second half of the follicular phase. In cycling, parous females, cortisol increases during the follicular phase, culminating with a peak around ovulation. Cortisol titers then decrease fairly quickly at the beginning of the luteal phase (as progesterone concentrations are rising), and remain low throughout most of the luteal phase (Fig. 4).

Our findings correspond with results from a growing number of species, which show that GC expression increases before ovulation (e.g. musk shrews (10), humans (38), pandas (39), and sheep (40)). The most detailed studies have been conducted in rats, and have shown that this increase is not due to overall upregulation of adrenal activity, but is due to a change in the amplitude of the circadian rhythm (6, 38). Specifically, the morning peak in corticosterone is highest preceding ovulation (proestrus) and lowest immediately following ovulation, but nadir values remain the same. This change in GC expression appears to play a critical role in the promotion of ovarian function, because suppression of adrenal activity or disruption of GC cycles is associated with ovarian dysfunction (4, 9, 11, 12). Furthermore, in humans and rats, the administration of exogenous GCs can increase the success of IVF and embryo transfer (3, 41). Therefore, it is not surprising that GCs may play a similarly positive role in reproductive function in elephants.

Previous studies of cortisol and estrous cycles in elephants have yielded mixed results. In a large study of African and Asian elephants, Brown *et al*. (21) reported that stage of estrous (follicular or luteal) did not have a significant effect on cortisol titers. Similarly, Oliveira *et al*. (23) found that mean cortisol values in Asian elephants were not significantly different between follicular and luteal phases; however, they also reported that cortisol titers were significantly higher in cycling females than in pregnant females. They suggest this difference is due to higher stress in cycling females (caused by increased attention from males), but do not discuss a possible role for cortisol in ovarian function. Bechert *et al*. (18) found a significant negative relationship between progesterone and cortisol in African elephants, but dismissed the relationship as lacking biological significance. One of the challenges in dissecting the relationship between progesterone and cortisol is that the cycles are not completely off-set (i.e. they are only 130° off-set, as opposed to 180°). As a consequence, the correlation between these two hormones is muted, and the difference in average cortisol titers between follicular and luteal phases is dampened. Furthermore, since adrenal activity is influenced by many factors, cortisol cycles have more associated variability compared with progesterone cycles, making it more difficult to detect cyclic fluctuations in cortisol expression. Time series analysis provides a more detailed understanding of hormone patterns. Our results help to resolve the conflicting reports in the literature and suggest that results that were previously dismissed should be given further consideration.

### Cortisol patterns in nulliparous females

Compared with parous females, we found that females who failed to conceive exhibited relatively high cortisol titers during the luteal phase (Fig. 5). In humans, females who have elevated cortisol levels during the mid-luteal phase are more likely to miscarry (42). In mice, exposure to a stressor (increased corticosterone) during early pregnancy causes a significant reduction in implantation rates, which appears to be mediated through changes in E_2_ (43). Therefore, although a peak in cortisol at the end of the follicular phase may be necessary to support ovulation (see above), low levels of cortisol during the luteal phase may be important for implantation and maintenance of a pregnancy. Disruption of these fluctuations may contribute to reproductive failure in elephants. In addition to our results, comparisons of cycling and non-cycling elephants have yielded some evidence which suggests that dampened cortisol patterns may be associated with ovarian dysfunction. Although mean cortisol values do not differ significantly between cycling and non-cycling females (21, 24), the within-individual range of cortisol expression for cycling Asian elephants (mean range±s.e.m.: 90.5±31.1 ng/ml) is nearly double the range for non-cycling females (47.7±18.3 ng/ml) (21).

### Proposed role for cortisol

Elephants exhibit several unique characteristics of ovarian activity compared with other mammals (15, 28, 29). One of the most notable differences is that there are two waves of follicle development during the follicular phase. Both waves culminate with an increase in 17β-E_2_ and an LH peak that are indistinguishable. However, ovulation only occurs after the second LH peak. Recent evidence has suggested that the purpose of the first follicular wave is to produce accessory corpora lutea (acCLs), which become the primary source of inhibin and promote ovulation of a single dominant follicle after the second LH peak (28). However, the mechanisms driving the different outcomes of the two follicular waves remain unknown.

GCs have been shown to play an important role in the regulation of ovarian function and synchronization of reproductive events in mice, rats, and humans (reviewed in (2, 3, 4, 5, 44)). They contribute to follicle maturation, ovulation, luteinization, and healing of the rupture site. They can also induce female sexual behavior (10). These permissive or stimulatory effects of cortisol should not be overlooked in studies of reproductive function. Furthermore, it is important to consider that the actions of GCs are only partially mediated by circulating concentrations; they are also influenced by locally expressed enzymes that serve to activate or inactivate GCs (11β-hydroxysteroid dehydrogenase (11βHSD) 1 and 2 respectively) (5) and background concentrations of other hormones, particularly estrogens (4).

We propose that cortisol helps to coordinate important events throughout the estrous cycle through a combination of agonistic and antagonistic effects. During the first follicular wave, intermediate cortisol titers may be insufficient to support ovulation (4), and may also prevent ovulation by inhibiting the expression of LH receptors (5). During the second follicular wave, the relatively high cortisol levels (particularly the peak at the end) may promote ovulation (4). This corresponds with the findings that acute stressors can stimulate ovulation (1). Local ovarian concentrations of cortisol may be further augmented via 11βHSD1, which activates cortisol (5). Cortisol may also contribute to the luteinization of the acCLs (3, 5), which are produced during the first follicular wave and start to luteinize during the second follicular wave (28). We would therefore predict that in non-cycling elephants, pre-ovulatory levels of cortisol might be lower than in cycling females, thereby inhibiting ovulation. If this is the case, then administration of exogenous GCs may help to stimulate ovulation. Indeed, treatment with exogenous GCs has been shown to increase oocyte production and quality in humans and rats (3, 41).

During the luteal phase, low cortisol concentrations may be required for proper implantation and luteal function (42, 45). This corresponds with a previous finding that cortisol titers are significantly lower in pregnant elephants than in cycling elephants (23). Increased expression of 11βHSD2 (dehydrogenase), an enzyme which inactivates cortisol, may further decrease local cortisol levels (5). The antagonistic effects of cortisol at this stage may serve to integrate information about external conditions and provide a ‘last-ditch’ mechanism to regulate reproduction. In elephants that exhibit regular estrous cycles but fail to conceive, pharmacological suppression of cortisol during the luteal phase may facilitate pregnancy.

## Conclusions

Integrating both permissive and suppressive effects of cortisol in the studies of ovarian and luteal function is important for understanding the complexity of endocrine interactions. Although the suppressive effects of chronically elevated GCs are important, efforts to understand reproductive dysfunction should not ignore the permissive effects of GCs under normal physiological levels. Elephants provide a unique opportunity to study adrenal–ovarian interactions because they have two LH peaks with different functions during the follicular phase. Understanding how cortisol mediates the differences between these LH peaks will offer valuable insights about the effect of cortisol on ovarian function. Furthermore, it may highlight possible solutions for resolving the fertility problems in captive elephants.

## Author contribution statement

K V Fanson and T Keeley conducted all endocrine assays. B G Fanson performed the statistical analysis. All authors contributed to the project design and manuscript preparation and have approved the final manuscript.

## Figures and Tables

**Table 1:** List of Asian elephants included in the study.

**ID**	**Institution**	**Age** (years)	**Successful pregnancy?**	**Number of cycles** (number of years)
F1	Melbourne Zoo	13	Yes (AI)	7 (1.7)
F2	Melbourne Zoo	6	Yes (AI)	9 (2.4)
F3	Melbourne Zoo	33	No (natural)	6 (1.5)
F4	Melbourne Zoo	5	Yes (AI)	8 (2.0)
F5	Taronga Zoo	14	Yes (natural)	10 (2.4)
F6	Taronga Zoo	14	Yes (AI)	7 (1.8)
F7	Taronga Zoo	7	No (both)	21 (5.7)
F8	Taronga Zoo	9	Yes (natural)	5 (1.1)

‘Age’ represents an individual's age at the start of the study (2006). The type of insemination (natural mating or artificial insemination (AI)) is indicated in parentheses in the pregnancy column. ‘Number of cycles’ indicates the number of estrous cycles analyzed for each female.

**Table 2:** Statistical results from time series regression models fitting a sinusoidal wave. For each hormone, sin() and cos() jointly define the sinusoidal wave and if either parameter is significant, then there is statistical support for a cyclic pattern. *R* ^2^ values indicate how well the model fit the data for each hormone. Coherency values indicate the strength of the association between progesterone and cortisol (i.e. similar cycle frequency). Bold indicates significant values (*P*<0.05).

**ID**	**Progesterone**			**Cortisol**			**Coherency**
sin()	cos()	*R* ^2^	sin()	cos()	*R* ^2^
F1	**0.107**	**0.204**	0.76	**−0.157**	**−0.102**	0.30	**0.47**
F2	−0.014	**−0.478**	0.80	0.062	0.024	0.03	0.12
F3	**−0.100**	−0.037	0.61	0.076	−0.104	0.06	0.13
F4	**0.127**	**−0.235**	0.79	0.025	**0.070**	0.08	**0.27**
F5	**−0.246**	**0.215**	0.78	0.037	**−0.094**	0.13	**0.30**
F6	**0.086**	−0.037	0.56	0.052	0.055	0.17	**0.45**
F7	−0.027	**0.149**	0.61	−0.034	**−0.069**	0.18	**0.39**
F8	**0.226**	−0.016	0.74	**−0.083**	**0.078**	0.31	**0.62**
